# COVID-19 Lockdown and Lifestyle Changes in Saudi Adults With Types 1 and 2 Diabetes

**DOI:** 10.3389/fpubh.2022.912816

**Published:** 2022-07-08

**Authors:** Nasser M. Al-Daghri, Abeer A. Almiman, Kaiser Wani, Malak N. K. Khattak, Naji J. Aljohani, Hanan Alfawaz, Abdulaziz Al-Hameidi, Dara Aldisi, Ghadah Alkhaldi, Shaun Sabico

**Affiliations:** ^1^Chair for Biomarkers of Chronic Diseases, Biochemistry Department, College of Science, King Saud University, Riyadh, Saudi Arabia; ^2^Department of Medicine, Obesity, Endocrine, and Metabolism Center, King Fahad Medical City, Riyadh, Saudi Arabia; ^3^Department of Food Science and Nutrition, College of Food Science and Agriculture, King Saud University, Riyadh, Saudi Arabia; ^4^Saudi Diabetes Charity, Riyadh, Saudi Arabia; ^5^Department of Community Health Sciences, College of Applied Medical Sciences, King Saud University, Riyadh, Saudi Arabia

**Keywords:** COVID-19, diabetes, SARS-CoV-2, lifestyle, diet, physical activity

## Abstract

**Objective:**

We aimed to evaluate and compare the impact of COVID-19 lockdown on lifestyle changes and other common related effects of the lockdown in Saudi adults with diabetes mellitus (DM), both type 1 (T1D) and type 2 diabetes (T2D).

**Methods:**

265 T1D and 285 T2D individuals were included in this cross-sectional survey during lockdown using an online questionnaire and compared with 297 participants without DM. Variables included demographics, treatment changes, use of supplements, change in sleeping habits and physical activity, dietary changes, social and mental health, and education and awareness during COVID-19 lockdown.

**Results:**

The COVID-19 lockdown was associated with more treatment doses in people with T1D but not in those with T2D (*p* = 0.003). More participants with T1D and T2D than the control group reported that they felt symptoms of depression during lockdown (ORs of 1.83, *p* = 0.008 and 2.2, *p* = 0.001, respectively) and that lockdown affected them psychologically (ORs of 1.64, *p* = 0.019 and 1.85, *p* = 0.005, respectively). More participants with T1D than controls reported that their physical activity decreased during lockdown (OR of 2.70, *p* = 0.024). Furthermore, significantly lesser participants in both DM groups than controls agreed that the health education regarding COVID-19 covered everything (ORs of 0.41, *p* < 0.001 and 0.56, *p* < 0.001, respectively for T1D and T2D groups). Regarding dietary habits, the DM groups reported more changes in either the number of daily meals, meal content, or mealtimes than the control group.

**Conclusions:**

COVID-19 lockdown-associated lifestyle changes were more prevalent in individuals with T1D and T2D compared to control. Findings may assist public health authorities in outlining their responses in pandemics and promote healthy lifestyle adaptations in this high-risk cohort to limit adverse effects in future lockdowns.

## Introduction

In December 2019, Wuhan became the first city in China to record some unidentified pneumonia cases, which was later declared a global pandemic, referred to as the COVID-19 pandemic, by the World Health Organization (WHO) (1). A novel coronavirus called the Severe Acute Respiratory Syndrome Coronavirus 2 (SARS-CoV-2) was responsible for this pandemic (2). The pandemic had an unprecedented impact on global health and is responsible for more than 6.1 million human deaths at the time of writing this manuscript (3). Coronaviruses belong to the well-studied Coronaviridae family with a single-stranded positive-sense RNA genome surrounded by an extracellular membrane that contains a case of spike glycoproteins (4, 5). Coronavirus strains known for their lower respiratory tract severe disease characteristics include SARS-CoV (severe acute respiratory syndrome), MERS-CoV (Middle East respiratory syndrome), and the recently identified SARS-CoV-2 (COVID-19) (6–8).

By and large, the global population has had an unprecedented experience of the measures taken by the governments and the local authorities to mitigate the health impact of this pandemic (9). In the same essence, the Saudi authorities implemented a nationwide lockdown in multiple phases in the Kingdom of Saudi Arabia (KSA) on March 15, 2020, following the emergence of the COVID-19 pandemic (10). Though most cases report mild to moderate symptoms; however, those with an established risk of severe COVID-19 symptoms include elderly persons, especially with comorbidities including diabetes mellitus, cardiovascular diseases, and micronutrient deficiencies such as vitamin D deficiency (11–14). Diabetes mellitus (DM) is a chronic disease categorized by abnormally high blood glucose levels triggered by a deficiency in insulin action and/or insulin secretion, and its prevalence worldwide and in the Middle East is estimated to be around 536.6 million (10.5%) and 72.7 million (16.2%), respectively (15). DM usually raises the risk for infectious severity and susceptibility due to its deleterious effects on host immunity (16). Therefore, individuals with diabetes are at a higher risk of viral and bacterial infections resulting in hospitalization and mortality.

Consequently, poorly controlled DM is a risk factor for several infectious diseases (17). Furthermore, the highly proactive measures taken during the pandemic contributed to fear, anxiety and/or depression in the general population (18–20). The pandemic lockdowns during the year 2020 affected the lifestyle of everybody in general and individuals in the high-risk group in particular. There are reports of increased feelings of stress or anxiety due to the lockdown in this group (21). In Saudi Arabia, multiple studies reported the effects of COVID-19 lockdown on the lifestyles of the population in general, with many highlighting changes in sleep hours, physical activity, diet, stress and anxiety levels, etc. (22–25).

The research related to COVID-19 infection and the effects of resulting lockdowns is a rapidly evolving field at a global level. Several studies have reported the impact of COVID-19 on DM patients, implying that COVID-19 may increase the complications of the disease among those with DM (26, 27). COVID-19 complications in people with diabetes are generally caused by an imbalance in the angiotensin-converting enzyme 2 (ACE2) activation pathways, which leads to an inflammatory response. In the pancreas, this imbalance leads to acute-cell dysfunctionality and complications such as vasculopathy (28). As a result, individuals with diabetes were advised to take specific preventive measures during the pandemic lockdowns (29, 30). However, much is unknown about the dynamics of this disease, and more research to better understand the clinical determinants of disease severity can help improve patient care throughout the healthcare system (31).

It is reasonable to assume that COVID-19 lockdown and associated restrictions may have altered dietary habits, physical activity levels, mental wellbeing, treatment plans, ease of getting medicines and medical advice on time, and sleeping patterns in patients with DM (32, 33), however, there is limited data on such information, particularly in this region. As a result, the current study sought to assess the impact of the COVID-19 lockdown in KSA on individuals with type 1 (T1D) and type 2 (T2D) diabetes compared with the general population using various lifestyle and health parameters such as changes in physical activity habits and dietary patterns to identify high risk timely tailored parameters in the two DM cohorts.

## Materials and Methods

### Study Participants and Study Design

In KSA, an online electronic survey during the COVID-19 pandemic lockdown was conducted between March 23 and June 20, 2020. This survey was done in collaboration with the Saudi Charitable Association of Diabetes (SCAD), which provides healthcare services to the diabetes population in KSA. The population chosen for our study was the subscribers of SCAD from different regions (Northern, Center, Western, Eastern, and Southern) in KSA. Participants voluntarily joined in this survey, and individuals with diabetes all vary in the disease's progression and treatments. The inclusion criteria included adult Saudis who were in KSA during the lockdown and had access to the internet. In addition, those with previously diagnosed diabetes were categorized as T1D or T2D groups, and the rest of the respondents with no history of diabetes were categorized as the control group. There were no additional exclusion criteria other than failure to complete the questionnaire.

Ethical approval for this study was obtained from the Ethics Committee for Scientific Research and Post Graduate Studies at the College of Science, King Saud University (KSU), Riyadh, KSA. The online electronic survey also included the informed consent form, which had to be agreed upon by the respondents before participating in the survey.

### Questionnaire

A standardized questionnaire was adapted from earlier studies (23, 34–36), and the online link of the Arabic version of the questionnaire prepared was made available by SCAD to its subscribers for distribution through different social media platforms throughout KSA. The questionnaire used had three sections:

#### Section 1

This section included questions for getting demographic and anthropometric data, where information on participants' gender, age, weight, height, and the region was asked (information presented in Table 1).

**Table 1:** Anthropometric and demographic differences in the study groups.

	**Study groups**			***P*** **-value**		
**Parameter**	**Control**	**T1D**	**T2D**	**T1D vs. control**	**T2D vs. control**	**T2D vs. T1D**
***N***	**297 (92/205)**	**265 (117/148)**	**285 (177/108)**			
Age (years)	38.7 ±10.1	43.2 ± 15.0	52.3 ± 9.7	<0.001	<0.001	<0.001
BMI (kg/m^2^)	28.9 ± 8.7	26.9 ± 6.3	29.9 ± 5.6	0.004	0.50	<0.001
**Region**						
North	16 (5.4)	11 (4.2)	7 (2.5)			
Central	132 (44.4)	134 (50.6)	148 (51.9)			
West	41 (13.8)	55 (20.8)	65 (22.8)	<0.001	<0.001	0.76
East	58 (19.5)	51 (19.2)	49 (22.8)			
South	50 (16.8)	14 (5.3)	16 (5.6)			
**Occupation**						
In-person?	148 (49.8)	67 (25.3)	100 (35.1)	<0.001	<0.001	0.016
Working remotely?	148 (49.8)	108 (40.8)	97 (34)	0.034	<0.001	0.12
Retired?	18 (6.1)	65 (24.5)	114 (40.0)	<0.001	<0.001	<0.001

*Data presented as mean ± SD for continuous variables and frequency (%) for categorical variables. T1D and T2D represent type 1 and 2 diabetes groups, respectively. P < 0.05 is considered significant*.

#### Section 2

This section included information on COVID-19 pandemic-related parameters like medication and treatment doses, micronutrient supplements and lifestyle habits, etc., during the lockdown were asked through yes/no questions (information presented as Table 2).

**Table 2:** Differences in responses to yes/no questions in the study groups.

	**Study groups**			***P*** **-value**		
**Parameter**	**Control**	**T1D**	**T2D**	**T1D vs. control**	**T2D vs. control**	**T2D vs. T1D**
***N***	**297 (92/205)**	**265 (117/148)**	**285 (177/108)**			
**DM treatment during pandemic**						
Taking medication		249 (94.0)	267 (93.7)	—	—	0.52
Insulin		199 (75.1)	88 (30.9)	—	—	<0.001
Pills		96 (36.2)	248 (87.0)	—	—	<0.001
DM management plan changed during pandemic?		34 (12.8)	38 (13.3)	—	—	0.48
Treatment doses increased during pandemic?		52 (19.6)	27 (9.5)	—	—	0.003
Treatment doses decreased during pandemic?		28 (10.6)	30 (10.5)	—	—	0.91
Whether respondents on diet management?	88 (43.1)	42 (15.8)	55 (19.3)	<0.001	<0.001	0.31
Whether respondents on weight loss management?	127 (42.8)	36 (13.6)	48 (16.8)	<0.001	<0.001	0.020
On diet + weight loss management?	108 (46.6)	55 (20.8)	58 (20.4)	<0.001	<0.001	0.68
**Supplement use**						
Vitamin D?	150 (50.5)	103 (38.9)	122 (42.8)	0.004	0.038	0.20
Vitamin C?	201 (67.7)	151 (57.0)	171 (60.0)	0.006	0.033	0.26
Zinc?	119 (40.1)	55 (20.8)	66 (23.2)	<0.001	<0.001	0.28
**Psychological changes**						
Insomnia?	121 (40.7)	99.4 (37.4)	107 (37.5)	0.43	0.44	0.52
Depression?	62 (20.9)	47 (17.7)	49 (17.2)	0.20	0.15	0.48
**Sleeping habits**						
Sleeping hours increased during Covid-19?	129 (43.4)	122 (46.0)	103 (36.1)	0.55	0.08	0.02
Was your sleep continuous?	133 (44.8)	121 (45.7)	124 (43.5)	0.86	0.80	0.61
Do you take sleeping pills?	23 (7.7)	9 (3.4)	14 (4.3)	0.030	0.18	0.40
**Physical activity**						
Did exercise increase during lockdown?	118 (39.7)	102 (38.5)	81 (28.4)	0.79	0.005	0.014
Did exercise decrease during lockdown?	128 (43.1)	97 (36.6)	134 (47.0)	0.12	0.36	0.015
Walking?	194 (65.3)	171 (64.5)	184 (64.6)	0.87	0.86	0.99
Cycling?	21 (7.1)	22 (8.3)	20 (7.0)	0.63	0.98	0.63
Home sports?	159 (53.5)	137 (51.7)	96 (33.7)	0.67	<0.001	<0.001
Swimming?	23 (7.7)	29 (10.9)	22 (7.7)	0.24	0.99	0.24
**Length of exercise during Covid-19**						
<10 min?	82 (27.6)	58 (21.9)	91 (31.9)!	0.12	0.28	0.009
10–30 min?	87 (29.3)	80 (30.2)	69 (24.2)	0.85	0.19	0.13
30–45 min?	29 (9.8)	34 (12.8)	27 (9.5)	0.29	0.90	0.22
46–60 min?	22 (7.4)	26 (9.8)	32 (11.2)	0.36	0.12	0.68
More than 60 min?	6 (2.0)	11 (4.2)	9 (3.2)	0.22	0.44	0.65
**Methods preferred in health education**						
Remote communication?	140 (47.1)	100 (37.7)	85 (29.8)			
Social media?	129 (43.4)	141 (52.2)	177 (62.1)	0.06	<0.001	0.10
Others?	28 (9.4)	24 (9.1)	23 (8.1)			
**Entities followed for COVID-19 information**						
SCAD?	90 (30.3)	45 (17.0)	40 (14.0)			
YouTube?	81 (27.3)	60 (22.6)	90 (31.6)	<0.001	<0.001	0.06
Other associations?	26 (8.8)	35 (13.2)	25 (8.8)			
Others?	100 (33.7)	125 (47.2)	130 (45.6)			
**Were you diagnosed with COVID-19?**	41 (13.8)	41 (15.5)	44 (15.4)	0.63	0.64	0.99
**If yes, did you experience**						
Fever?	14 (34.1)	16 (39.0)	19 (43.2)	0.82	0.51	0.83
Cough?	11 (26.8)	15 (36.6)	11 (25.0)	0.48	0.85	0.35
Loss of taste and smell?	11 (26.8)	12 (29.3)	16 (36.4)	0.81	0.36	0.50
Shortness of breath?	7 (17.1)	5 (12.2)	12 (27.3)	0.76	0.31	0.11
Throat pain?	7 (17.1)	11 (26.8)	16 (36.4)	0.42	0.05	0.36

*Data presented as frequency (%). T1D and T2D represent type 1 and type 2 diabetes groups, respectively. P < 0.05 is considered significant*.

#### Section 3

This section had 16 closed format questions in the Likert scale divided into four sub-sections. It provided information about the level of agreement in “COVID-19 measures,” “dietary changes,” “changes in social and mental health,” and “awareness” among participants during COVID-19 lockdown derived from “4,” “6,” “3” and “3” questions respectively (information presented as Table 3). The scale used to calculate the level of agreement was “strongly agree,” “agree,” “neutral,” “disagree,” and “strongly disagree.”

**Table 3:** Differences in the levels of agreement according to COVID-19 lockdown.

**Questions**	**Control (297)**	**T1D (265)**	**T2D (285)**	***P*-value**
**COVID-19 measures during lockdown**						
1. Adherence to COVID-19 preventive measures?	271 (91.2)	254 (95.8)^*^	270 (94.7)	0.02
2. Did you maintain social distance?	225 (75.8)	176 (66.4)	187 (65.6)	0.06
3. Did your family maintain social distance?	206 (69.4)	187 (70.6)	168 (58.9)^$^	0.03
4. Do you think diabetes is a risk factor for corona?	195 (65.7)	171 (64.5)	179 (62.8)	0.43
Total score (Maximum 4)	3.18 ± 1.1	3.27 ± 0.9	3.11 ± 0.9	0.17
**Dietary changes during lockdown**						
5. Eating habits changed?	153 (51.5)	90 (34.0)^*^	99 (34.7)^*^	<0.001
6. Mealtimes changed?	148 (49.8)	82 (30.9)^*^	73 (25.6)^*^	<0.001
7. Number of meals changed?	146 (49.2)	81 (30.6)^*^	70 (24.6)^*^	<0.001
8. Meal content changed?	148 (49.8)	76 (28.7)^*^	84 (29.5)^*^	<0.001
9. More fast food intake?	196 (66.0)	168 (63.4)	171 (60.0)	0.68
10. Decrease in homemade food intake?	16 (5.4)	10 (3.8)	18 (6.3)	0.69
Total score (Maximum 6)	2.81 ± 1.8	2.0 ± 1.6^*^	1.90 ± 1.6^*^	<0.001
**Changes in social and mental health during lockdown**						
11. Relationship with family got affected?	18 (6.1)	10 (3.8)	11 (3.9)	0.37
12. Has lockdown affected you psychologically?	135 (45.5)	132 (49.8)	133 (46.7)	0.25
13. Felt symptoms of depression?	91 (30.6)	95 (35.8)	92 (32.3)	0.51
Total Score (Maximum 3)	1.01 ± 0.9	1.14 ± 0.9	1.10 ± 0.9	0.28
**COVID-19 education and awareness during lockdown**						
14. Do you think health awareness for Covid-19 was carried out previously?	225 (75.8)	151 (57.0)^*^	176 (61.8)^*^	<0.001
15. Did you make maximum use of health education?	226 (76.1)	146 (55.1)^*^	167 (58.6)^*^	<0.001
16. Do you think health education for Covid-19 covered all educational needs?	211 (71.0)	148 (55.8)^*^	173 (60.7)^*^	0.003
Total score (Maximum 3)	2.23 ± 1.1	1.68 ± 1.3^*^	1.81 ± 1.3^*^	<0.001

*Data presented as frequency (%) for categorical variables and mean ± SD for the total score in each section. T1D and T2D represent type 1 and 2 diabetes groups, respectively*.

* *and*

$ *represent the differences compared to the “control” and “T1D” groups. P < 0.05 is considered significant*.

The reliability of the Arabic version of the questionnaire (Supplementary File 1) was tested in a pilot study, also organized by the SCAD, on 50 samples. The reliability test in all four sub-sections of the Linkert scale section of the questionnaire achieved a Cronbach's alpha of >0.7 with an overall score of 0.84 for a total of 16 items (Supplementary Table S1).

### Data Analysis

Sample size calculation was done using Raosoft online. The sample size needed was calculated using an error margin of 2.5%, and the prevalence of T1D and T2D was 0.03 and 18.3%, respectively. To get a confidence level of 95%, a minimum of 500 sample size was set to achieve the study's objectives. All calculations and data analysis was done using SPSS version 16.5. The data in three study groups were compared using chi-square test for categorical variables and the independent student's *t*-test for continuous variables. The agreement levels for sections “COVID-19 measures,” “Dietary changes,” “Social and Mental Health during COVID-19 lockdown,” and “Health education and awareness” were presented as frequency (%), and an average value of scores for all the items in each section was presented as mean ± standard deviation.

Logistic regression analysis (univariate and multivariate) was run separately with T1D/T2D versus control as dependent variable and close-ended questions as independent variables. In the multivariate model, adjustment was made with age, gender, BMI, and demographic variables. A 95% confidence interval (95% CI) for the odds ratio (OR) was checked to evaluate significant independent categorical variables. Statistical significance was set at *p* < 0.05.

## Results

### Anthropometric and Demographic Differences Between the Study Groups

Table 1 shows the anthropometric and demographic differences among study groups. The subjects in the T2D group were significantly older and had higher BMI compared to both T1D and controls s (*p* < 0.001). Majority of all participants (about 50%) were from the central region. The control group also had the highest prevalence of employed participants (be it working remotely or in-person) than DM groups with a higher prevalence of “retired from work” participants.

### Differences in Responses to Yes/No Questions in the Study Groups

Table 2 shows the information obtained *via* yes/no questions. As expected, most DM groups took medications with higher proportions of insulin and oral hypoglycemic pills in the T1D and T2D groups, respectively (*p* < 0.001 for both). Dietary and weight loss management were overwhelmingly from the control group. The use of dietary supplements such as vitamins D, C, and zinc was highest in the control group (*p* < 0.05 for all). There was no difference in the use of supplements between the DM groups. When asked whether sleeping hours increased during the lockdown, less than half in all groups (43.4, 46, and 36.1% in control, T1D and T2D, respectively) said “yes,” and a small number indicated that they were on sleeping pills. Participants whose exercise decreased during lockdown were highest in the T2D group (47.0%). Those with <10 min/per day of exercise were higher in T2D than T1D participants (*p* = 0.009). When asked if they were infected with COVID-19, around 15% of study groups answered “yes.” There was no difference in the prevalence of opting for different COVID-19 symptoms experienced.

### Levels of Agreement for COVID-19-Related Questions

Table 3 shows the level of agreement of participants with respect to the COVID-19-related questions. The complete data for these sections in the questionnaire has been tabulated as Supplementary Table S2. The majority in all groups agreed that they followed COVID-19 preventive measures during the lockdown (>90% in each group). There was no difference statistically when the total score for all four items of the section “COVID-19 measures” was checked. More participants from the control group agreed that their eating habits changed, as well as mealtimes, meal content, and the number of meals than both diabetes groups (*p* < 0.001 for all). No difference was observed between groups related to changes in social and mental health issues during the lockdown. Lastly, the majority (>55%) agreed that health awareness campaigns by SCAD for COVID-19 were carried out previously. A lower proportion in both diabetes groups than control agreed that awareness campaigns covered all aspects of COVID-19-related information and that they maximized the information given (*p* < 0.01 in both).

### Multinomial Regression for Calculating the Odds Ratio of Lifestyle Changes in Diabetes vs. Control Groups

**Figure 1:**
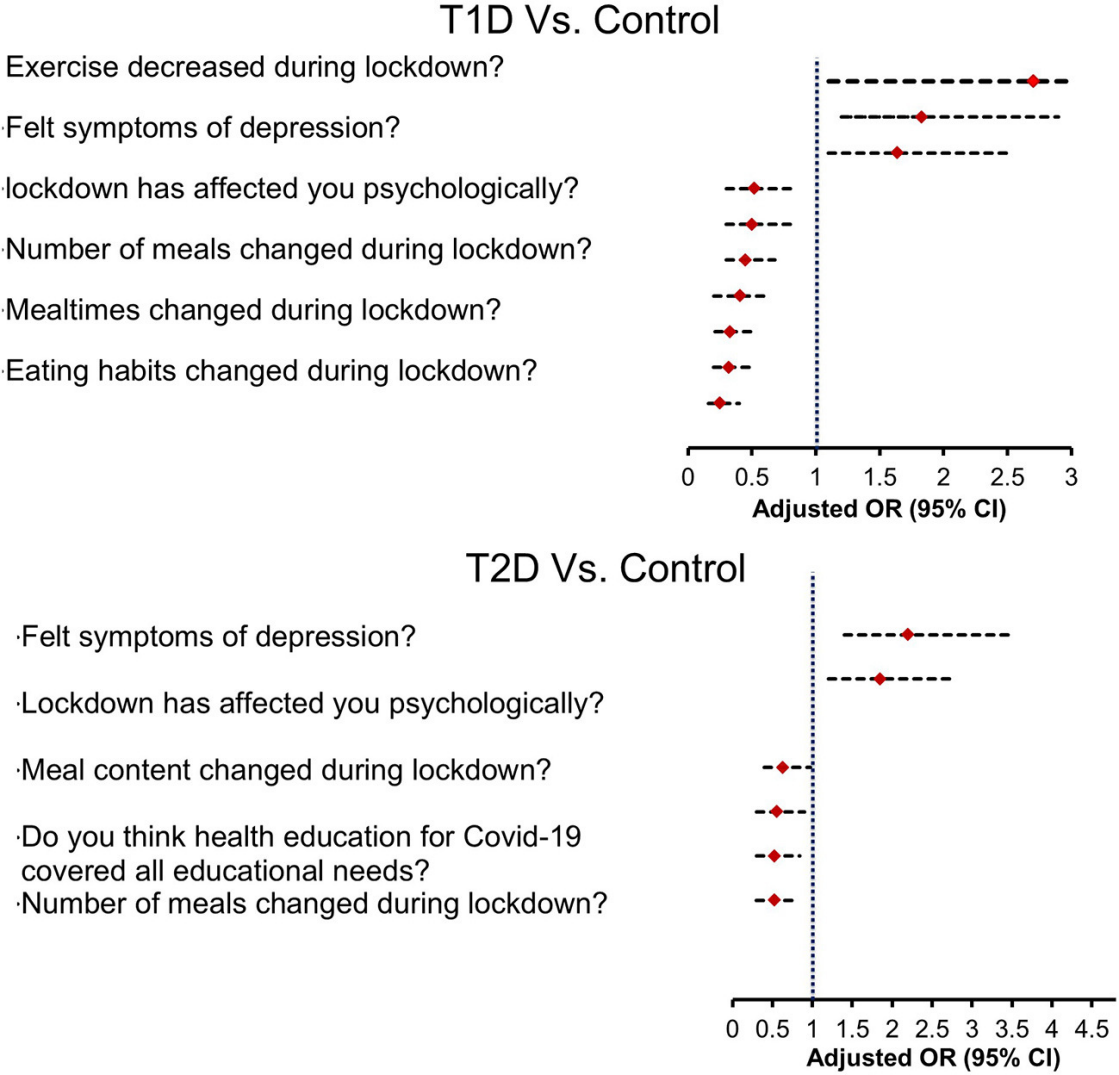
Forest plot depicting adjusted odds of responses related to lifestyle changes in diabetes vs. control groups.

Multinomial logistic regression was done to examine differences between DM and control groups in the responses. Only significant independent variables were plotted as a forest plot in Figure 1. Data of the logistic regression analysis, including OR, 95% confidence interval, and *p*-values, has been provided as Supplementary Table S3. The regression model was adjusted with the participants' age, sex, BMI, and demographic status to extract the multivariate odds plotted in the figure. The analysis showed that factors like decreased exercise, depression, and use of supplements showed greater odds in diabetes groups compared to the control groups. However, the dietary changes like changes in meal content, number of meals, and mealtimes during lockdown showed lesser odds in diabetes groups compared to the control group. Also, the DM groups felt a lack of sufficient COVID-19 related information and health awareness during the lockdown than the control group.

## Discussion

The consequences of COVID-19 lockdown on the lifestyle of the general population and high-risk groups have been the area of interest because of this pandemic's massive impact on humankind. However, less has been investigated primarily in this population, where there is a huge prevalence of diabetes. Therefore, the present study aimed to evaluate and compare lifestyle changes due to COVID-19 lockdown in a population of adult Saudis with T1D and T2D. The study suggests lifestyle changes in terms of dietary, physical activity, sleeping hours, etc., were more prevalent in diabetes groups compared to the control. Also, the DM groups reported higher prevalence of insufficient health education and awareness related to COVID-19. Besides, the self-assessed changes in the psychological status of the respondents, like insomnia and depression, were also reported by a sizable proportion in all groups.

Diabetes, a chronic disease, is highly prevalent in this part of the world (18.3% of adults according to the International Diabetes Federation) (37), and it imposes a significant burden at the individual, familial, community, and global levels. Compliance with medical treatment is one essential lifestyle component for those with diabetes, and complications caused by uncontrolled glucose levels may occur because of this non-compliance. Our study suggested that the treatment doses increased during lockdown for those with T1D than T2D. This might have resulted from increased uncontrolled glycemia in T1D patients during the lockdown because of less availability of insulin medications. The study by Magliah et al. (38) reported that 66% of T1D patients had difficulty obtaining at least one type of their insulin medication supply during the lockdown. Reports also suggested that COVID-19 lockdown negatively affected weight and glucose control in individuals with diabetes, mainly in patients on insulin (39). In this lockdown, the emergency services were prioritized, and routine care services, which form the core of managing chronic diseases like diabetes, were jeopardized, increasing the risk of associated complications. Furthermore, the COVID-19 lockdown affected other aspects of the pharmacological and non-pharmacological management of DM, including access to doctor consultation, counseling services, availability of recommended dietary items, social interaction, restricted physical activity, amongst others (40, 41).

A large proportion of participants indicated that they had insomnia (>35%) and irregular sleep (>55%) during the lockdown. Besides, fewer subjects in the T2D group reported that the sleeping hours increased during lockdown compared to the T1D group. These results align with multiple studies that reported an increase in insomnia during COVID-19 lockdown (42–44). The primary cause for this has been the fear of getting infected, the economy-related stress, the social isolation and changes in social rhythms (44, 45). Sleep quantity and quality, though important in the general population, are more critical in managing diseases like diabetes, which require a multidimensional approach including adequate and time-sensitive quality sleep, a lack of which may increase diabetes severity and cardiovascular comorbidities (46, 47). Lack of proper sleep may also aggravate anxiety and depression, and a sizable proportion, even though self-assessed, in all three groups (>45%), agreed that the lockdown affected them psychologically. Therefore, an integrated multidimensional approach, including counseling and training on self-care behaviors, may be needed to mitigate the mental health decline in individuals with diabetes during the COVID-19 lockdown (48, 49).

Physical activity and exercise rates decreased during the lockdown in all groups. Apart from the glycemia-controlling medicines, exercise training programs especially aerobic exercises, have emerged as a useful tool in managing diabetes (50). In line with the results of our study, many recent studies highlighted a significant increase in physical inactivity in both the general population and in individuals with chronic diseases during lockdown (51). Studies indicated that lockdown measures were associated with significantly reducing physical activity levels in chronic patients like those on hemodialysis (52). Others, like one from the United Kingdom, reported a significant decrease in physical activity during this lockdown in the general population (53). Simple physical activity regimens developed and promoted to the public may help mitigate this problem in future pandemics (54).

The dietary guidelines announced by the World Health Organization to boost immunity during the COVID-19 outbreak included taking a balanced diet with the recommended amount of macro and micronutrients; however, the literature suggests that the lockdown resulted in less consumption of fresh produce and more consumption of comfort foods (55). This may have both short and long-term health effects. Earlier reports from Italy also reported that 46.1% of the study participants felt they ate more during the lockdown, particularly high-calorie foods such as chocolate, ice cream, and desserts (56). In this study, more individuals reported changes in eating habits, mealtimes, meal quantity, and quality in the control group compared to the diabetes groups. Sedentary behaviors, including increased screen time, prevalent during confinement, may directly be associated with an increase in unhealthy dietary patterns in lockdown (57). While important in the general population, good dietary practices are a prerequisite for managing the HbA1c levels in diabetes (58). Thus, stakeholders involved in diabetes care must conduct interventions aimed at increasing awareness in diabetes patients about appropriate self-care.

The results of this study could help health authorities and diabetes organizations like SCAD, which provide health care services to people with diabetes in Saudi Arabia, better handle these services in the event of a pandemic. According to the American Diabetes Association, conventional diabetes care for patients involves glucose management and monitoring, timely advice on food and physical exercise, availability, and distribution of medicines, and efforts to prevent infection in high-risk populations. This lockdown taught us a lot about how to better handle a future pandemic by showing us what not to do when it comes to access and availability of glycemic monitoring supplies like glucose strips, glucometers, and needles, among other things. In the event of future pandemics, findings of this study on dietary supplements may assist DM management organizations in emphasizing the need to focus on logistics to ensure that there are enough dietary supplements available and that these supplements are disseminated to patients. Participants in both DM groups believed that COVID-19 information was insufficient, which might assist the authorities in creating better lifestyle interventions for diabetics and other high-risk persons in future pandemics. The Findings on depression and the psychological effects of lockdown in diabetics may also help these organizations develop solutions for mental health, anxiety levels, the significance of enough sleep, and so on in high-risk individuals.

The strengths of the study included a relatively large number of respondents and the subjects recruited from different geographical regions of KSA. To the best of the authors' knowledge, this study is the first report of lifestyle changes during COVID-19 lockdown in both T1D and T2D Saudi subjects. At the same time, the authors would like to acknowledge some limitations which should be considered in interpreting the study findings. Firstly, this study was cross-sectional, which might introduce a potential risk of recall bias. However, our study intended to be a comparison analysis between the study groups. Secondly, participants in this study self-reported lifestyle changes during lockdown with no specific personality assessment like ones for psychological statuses like insomnia and depression, and they might have underestimated these changes. Thirdly, members of SCAD were recruited as participants for the study, which might have had some bias in the socio-economic class of overall Saudi subjects. Nevertheless, the study revealed some important findings about lifestyle changes in DM individuals during pandemic. The findings may help the concerned authorities and organizations prioritize interventions to handle DM management in future lockdowns.

## Conclusions

Our results show that the lockdowns imposed as restrictive measures against COVID-19 negatively affected the lifestyle and daily habits of Saudi individuals with DM. The present findings should help public health authorities and stakeholders involved in diabetes care to plan for future pandemics and promote healthy lifestyle changes in high-risk populations during lockdowns.

## Data Availability Statement

The original contributions presented in the study are included in the article/Supplementary Material, further inquiries can be directed to the corresponding author/s.

## Ethics Statement

The studies involving human participants were reviewed and approved by Ethics Committee of the Post-Graduate Studies, College of Science, King Saud University. The patients/participants provided their written informed consent to participate in this study.

## Author Contributions

NA-D, AA, and SS designed the study. DA and GA worked on the methodology. MK did the statistical analysis. AA, HA, AA-H, and DA helped in the data curation. AA and KW wrote the first draft. NA-D, HA, and SS revised the manuscript. NA-D, NA, and HA did the study supervision. All authors contributed and approved the final version of the paper.

## Funding

This research was funded by the Deanship of Scientific Research, King Saud University for funding through Vice Deanship of Scientific Research Chairs, Chair for Biomarkers of Chronic Diseases.

## Conflict of Interest

The authors declare that the research was conducted in the absence of any commercial or financial relationships that could be construed as a potential conflict of interest.

## Publisher's Note

All claims expressed in this article are solely those of the authors and do not necessarily represent those of their affiliated organizations, or those of the publisher, the editors and the reviewers. Any product that may be evaluated in this article, or claim that may be made by its manufacturer, is not guaranteed or endorsed by the publisher.
