# Supplementary material for: COVID-19 Lockdown and Lifestyle Changes in Saudi Adults With Types 1 and 2 Diabetes
Source: Front Public Health. 2022 Jul 8;10:912816. doi: 10.3389/fpubh.2022.912816 (PMC9304975; doi:10.3389/fpubh.2022.912816)
Supplement: Supplementary file 1 [file Table_1.DOCX]

Supplementary Material

**Supplementary table ST1.** Cronbach's alpha for the reliability of the questionnaire in the pilot study.

| **Questionnaire type** | **No of items** | **Cronbach Alpha** |
| --- | --- | --- |
| 1.       COVID-19 Measures | 4 | 0.719 |
| 2.       Dietary Changes During Pandemic | 6 | 0.791 |
| 3.       Social and Mental Health during COVID-19 | 3 | 0.879 |
| 4.       Health education and awareness | 3 | 0.761 |
| Overall response (Likert scale) | 16 | 0.836 |

**Supplementary table ST2.** Responses to Likert scale questions in the survey.

| **Questions** | **Agree** | **Neutral** | **Disagree** | | | | **p-Value** |
| --- | --- | --- | --- | --- | --- | --- | --- |
| **COVID-19 Measures** | | | | | | | |
| **1. Adherence to COVID-19 preventive measures?**  Control  T1DM**  T2DM* | 271 (91.2)  254 (95.8)  270 (94.7) | 18 (6.1)  7 (2.6)  15 (5.3) | 8 (2.7)  4 (1.5)  0 (0.0) | | | | 0.021 |
| **2.Your family maintain physical distance?**  Control  T1DM**  T2DM* | 206 (69.4)  187 (70.6)  168 (58.9) | 71 (23.9)  59 (22.3)  85 (29.8) | 20 (6.7)  19 (7.2)  32 (11.2) | | | | 0.025 |
| **3. Did you maintain social Distance**  Control  T1DM**  T2DM* | 225 (75.8)  176 (66.4)  187 (65.6) | 49 (16.5)  64 (24.2)  67 (23.5) | 23 (7.7)  25 (9.4)  31 (10.9) | | | | 0.06 |
| **4.Do you think Diabetes is a risk Factor for corona?**  Control  T1DM**  T2DM* | 195 (65.7)  171 (64.5)  179 (62.8) | 80 (26.9)  65 (24.5)  72 (25.3) | 22 (7.4)  29 (10.9)  34 (11.9) | | | | 0.43 |
| **Dietary Changes During Pandemic** | | | | | | | |
| **5. Eating habits changed?**  Control  T1DM**  T2DM** | 153 (51.5)  90 (34.0)  99 (34.7) | 79 (26.6)  85 (32.1)  79 (27.7) | | 65 (21.9)  90 (34.0)  107 (37.5) | | <0.001 | |
| **6. Mealtimes changed?**  Control  T1DM**  T2DM** | 148 (49.8)  82 (30.9)  73 (25.6) | 66 (22.2)  71 (26.8)  91 (31.9) | | 83 (27.9)  112 (42.3)  121 (42.5) | | <0.001 | |
| **7.Number of meals changed?**  Control  T1DM**  T2DM** | 146 (49.2)  81 (30.6)  70 (24.6) | 74 (24.9)  76 (28.7)  81 (28.4) | | 77 (25.9)  108 (40.8)  134 (47.0) | | <0.001 | |
| **8.Meal content changed?**  Control  T1DM**  T2DM** | 148 (49.8)  76 (28.7)  84 (29.5) | 77 (25.9)  82 (30.9)  82 (28.8) | | 72 (24.2)  107 (40.4)  119 (41.8) | | <0.001 | |
| **9.More fast food intake?**  Control  T1DM  T2DM | 196 (66.0)  168 (63.4)  171 (60.0) | 45 (15.2)  42 (15.8)  50 (17.5) | | 56 (18.9)  55 (20.8)  64 (22.5) | | 0.68 | |
| **10.More homemade food intake?**  Control  T1DM  T2DM | 255 (85.9)  235 (88.7)  242 (84.9) | 26 (8.8)  20 (7.5)  25 (8.8) | | 16 (5.4)  10 (3.8)  18 (6.3) | | 0.69 | |
| **Social and Mental Health during COVID-19 lockdown** | | | | | | | |
| **11. Do you think your relationship with family improved?**  Control  T1DM  T2DM* | 222 (74.7)  190 (71.7)  208 (73.0) | 57 (19.2)  65 (24.5)  66 (23.2) | | 18 (6.1)  10 (3.8)  11 (3.9) | 0.37 | | |
| **12. Do you think lockdown has affected psychologically?**  Control  T1DM  T2DM | 135 (45.5)  132 (49.8)  133 (46.7) | 59 (19.9)  64 (24.2)  67 (23.5) | | 103 (34.7)  69 (26.0)  85 (29.8) | 0.25 | | |
| **13. Felt symptoms of depression?**  Control  T1DM  T2DM | 91 (30.6)  95 (35.8)  92 (32.3) | 78 (26.3)  58 (21.9)  62 (21.8) | | 128 (43.1)  112 (42.3)  131 (46.0) | 0.51 | | |
| **Health education and awareness** | | | | | | | |
| **14. Do you think health awareness for Covid-19 was carried out previously?**  Control  T1DM**  T2DM** | 225 (75.8)  151 (57.0)  176 (61.8) | 54 (18.2)  73 (27.5)  73 (25.6) | | 18 (6.1)  41 (15.5)  36 (12.6) | | <0.001 | |
| **15. Did you make maximum use of health education?**  Control  T1DM**  T2DM** | 226 (76.1)  146 (55.1)  167 (58.6) | 53 (17.8)  77 (29.1)  82 (28.8) | | 18 (6.1)  42 (15.8)  36 (12.6) | | <0.001 | |
| **16. Do you think health education for Covid-19 covered all educational needs?**  Control  T1DM**  T2DM* | 211 (71.0)  148 (55.8)  173 (60.7) | 70 (23.6)  86 (32.5)  86 (30.2) | | 16 (5.4)  31 (11.7)  26 (9.1) | | 0.003 | |

**Note:** * denotes significance compared to control; ** denotes significance compared to control and another DM group; significance at p<0.05.

**Supplementary table ST3:** Multinomial Regression for odds ratio of lifestyle changes in diabetic groups compared to control group.

| **Parameters** | **Model 1 (Univariate)** | | **Model 2 (Multivariate)** | |
| --- | --- | --- | --- | --- |
|  | **OR (95% CI)** | **p** | **OR (95% CI)** | **p** |
| **T1D Vs. Control group** | | | | |
| Exercise decreased during lockdown? | 1.60 (0.8-3.2) | 0.16 | 2.70 (1.1-6.2) | 0.024 |
| Felt symptoms of depression? | 1.27 (0.9-1.8) | 0.19 | 1.83 (1.2-2.9) | 0.008 |
| lockdown has affected you psychologically? | 1.19 (0.9-1.7) | 0.3 | 1.64 (1.1-2.5) | 0.019 |
| Number of meals changed during lockdown? | 0.46 (0.32-0.64) | <0.001 | 0.52 (0.3-0.83) | 0.003 |
| Mealtimes changed during lockdown? | 0.45 (0.3-0.64) | <0.001 | 0.50 (0.3-0.80) | 0.001 |
| Eating habits changed during lockdown? | 0.48 (0.3-0.68) | <0.001 | 0.45 (0.3-0.68) | <0.001 |
| Do you think health education for Covid-19 covered all educational needs? | 0.52 (0.3-0.7) | <0.001 | 0.41 (0.2-0.6) | <0.001 |
| Meal content changed during lockdown? | 0.41 (0.3-0.61) | <0.001 | 0.33 (0.21-0.5) | <0.001 |
| Do you think health awareness for Covid-19 was carried out previously? | 0.42 (0.3-0.6) | <0.001 | 0.32 (0.2-0.5) | <0.001 |
| Did you make maximum use of health education? | 0.39 (0.3-0.6) | <0.001 | 0.25 (0.16-0.4) | <0.001 |
| **T2D Vs. Control group** | | | | |
| Felt symptoms of depression? | 1.08 (0.7-1.5) | 0.67 | 2.2 (1.4-3.5) | 0.001 |
| lockdown has affected you psychologically? | 1.05 (0.8-1.5) | 0.77 | 1.85 (1.2-2.8) | 0.005 |
| Meal content changed during lockdown? | 0.42 (0.30-0.59) | <0.001 | 0.63 (0.4-0.98) | 0.039 |
| Do you think health education for Covid-19 covered all educational needs? | 0.63 (0.4-0.9) | <0.001 | 0.56 (0.3-0.9) | <0.001 |
| Number of meals changed during lockdown? | 0.34 (0.24-0.50) | <0.001 | 0.53 (0.3-0.84) | 0.006 |
| Mealtimes changed during lockdown? | 0.35 (0.2-0.49) | <0.001 | 0.53 (0.3-0.82) | 0.005 |
| Do you think health awareness for Covid-19 was carried out previously? | 0.52 (0.4-0.7) | <0.001 | 0.43 (0.3-0.7) | <0.001 |
| Did you make maximum use of health education? | 0.45 (0.3-0.6) | <0.001 | 0.32 (0.2-0.52) | <0.001 |

Note: The data was derived from a multinomial logistic regression analysis and presented as odds ratio (95% confidence interval) of answering the listed questions in diabetic groups compared to control group. The multivariate OR was calculated by adjustment with age, sex, BMI and demographic status. Only those with significant adjusted OR’s were presented in the table. P<0.05 was considered as significant.
